# Bevacizumab and Sinus Venous Thrombosis: A Literature Review

**DOI:** 10.7759/cureus.19471

**Published:** 2021-11-11

**Authors:** Vikash Jaiswal, Esha Jain, Gazala Hitawala, Hanyou Loh, Suyog Patel, Pawan Thada, Varsha Nandwana, Shreya Pandey, Jonathan Quinonez, Sidra Naz, Joel D Stein, Wilson Cueva

**Affiliations:** 1 Research and Academic Affairs, Larkin Community Hospital, South Miami, USA; 2 Medicine, American University of Antigua, St. John’s, ATG; 3 Internal Medicine, JC Medical Center, Orlando, USA; 4 Medicine, National University of Singapore, Singapore, SGP; 5 Medicine, B J Medical College, Ahmedabad, IND; 6 Research, Larkin Community Hospital, South Miami, USA; 7 Medicine, Lady Hardinge Medical College, Delhi, IND; 8 Neurology/Osteopathic Neuromuscular Medicine, Larkin Community Hospital, South Miami, USA; 9 Internal Medicine, Beth Israel Deaconess Medical Center, Harvard Medical School, Boston, USA; 10 Osteopathic Neuromusculoskeletal Manipulative Medicine, Family Medicine, Sports Medicine, Pain Medicine, Lake Erie College of Osteopathic Medicine Bradenton, Bradenton, USA; 11 Pain Mangement, Osteopathic Neuromusculoskeletal Manipulative Medicine, Sports Medicine, Larkin Community Hospital, South Miami, USA; 12 Neurology, Larkin Community Hospital, South Miami, USA

**Keywords:** neurology and critical care, pediatrics emergency, vegf inhibitor, glioma, cerebral sinus venous thrombosis, bevacizumab

## Abstract

Pediatric glioma treatment can be confounded by eloquent anatomical location and pathologic and genetic characteristics. Current literature suggests that the vascular endothelial growth factor (VEGF) inhibitor bevacizumab has been linked to enhancing disease control; however, its safety and effectiveness are unknown. Bevacizumab has been linked with an increased incidence of intratumoral hemorrhage as well as arterial and venous thromboembolism. A rare adverse effect of chemotherapeutic treatment with bevacizumab is sinus venous thrombosis (SVT), with only a few cases reported to date. This review highlights the pathophysiology of bevacizumab, its rare and life-threatening side effect of SVT, and future recommendations.

## Introduction and background

Gliomas and their variants

A glioma is a brain tumor that originates from the glial cell lineage and functions as a support for neurons. Types of glial cells include astrocytes, ependymal cells, and oligodendrocytes, which give rise to astrocytomas, ependymomas, and oligodendrogliomas, respectively. Astrocytomas may develop in the cerebrum or cerebellum. They occur in adults or children and differ significantly in severity. Glioblastoma multiforme is a highly malignant high-grade astrocytoma, while a pilocytic astrocytoma is a low-grade cerebellar glioma more commonly found in the pediatric population [1]. Ependymomas are a rare type of primary brain tumor and occur more often in the pediatric population. The most common location for pediatric brain tumors is the cerebellum, and obstructive hydrocephalus and drop metastases are common sequelae. Oligodendrogliomas are another rare type of primary brain tumor. They are more commonly seen in middle-aged males and tend to have a better prognosis than other gliomas [1].

In all cases, diagnosis of a glioma requires radiographic evidence in the form of computed tomography (CT) or magnetic resonance imaging (MRI), as well as histologic evidence following a biopsy of the tumor. Although treatment is grade-dependent, it commonly involves surgery and adjuvant therapies of chemotherapy and radiation therapy [1].

Bevacizumab

Bevacizumab injection is a transparent solution given intravenously at doses of 5, 7.5, 10, or 15 mg/kg [2]. In the 1980s, it was discovered that vascular endothelial growth factor (VEGF) was an essential factor that regulated normal and abnormal growth of blood vessels, which led to the discovery of bevacizumab as an anticancer agent [3].

Bevacizumab inhibits the binding of cell surface receptors to VEGF (binds more than 97%), reducing the blood supply to cancerous cells, increasing the apoptosis of cancerous cells, and helping chemotherapeutic drugs to work more efficiently. It is crucial to be aware of the unfavorable adverse effects of bevacizumab. These side effects can include high blood pressure leading to hypertension, abnormal bleeding and wound healing, perforations in the stomach, and thromboembolic events. When used chronically, bevacizumab has been shown to decrease left ventricular ejection fraction. If patients encounter some of these side effects, bevacizumab should be immediately stopped to reduce the complications associated with these side effects [4].

## Review

Mechanism of action of bevacizumab

Bevacizumab is a humanized monoclonal antibody (MAB) that acts by inhibiting the signal protein VEGF. Bevacizumab is the first MAB approved for widespread use by the US Federal Drug Administration (FDA). Its approximate molecular weight is 149 kD, and it has an acidic pH of 6.2. Compared to other VEGF inhibitors, bevacizumab displays the longest half-life [5]. The function of bevacizumab is unique as it exhibits anti-angiogenic properties. It functions by inhibiting the formation and growth of new blood vessels in the body, also known as angiogenesis [6]. VEGF is a potent growth factor that enhances the proliferation and survival of endothelial cells. Clinicians recognize VEGF to be an important target for treatment against cancer [2].

**Figure 1:**
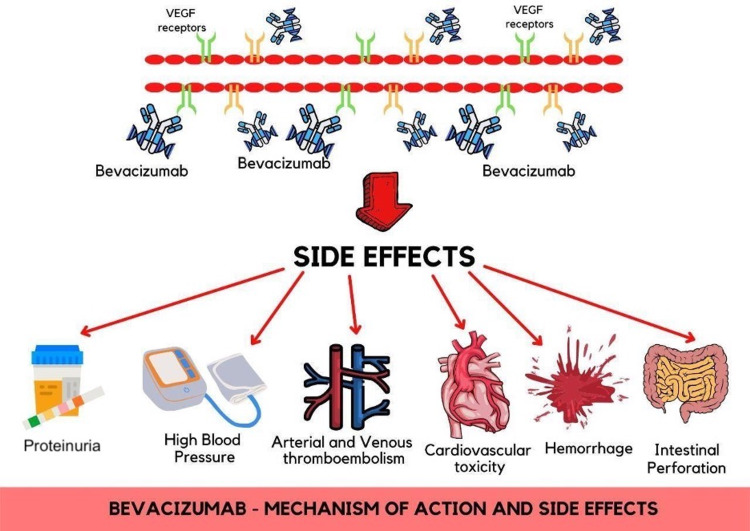
Mechanism of action of bevacizumab and its side effects associated with different organs in the human body. VEGF: vascular endothelial growth factor

VEGF plays a crucial role in enhancing the growth of various tumors in the human body. Especially in ongoing cancer states, VEGF is increasingly upregulated and produced. Due to the overexpression of VEGF in cancer states, VEGF is implicated in the progression of tumors. When found in high levels in the body, it is associated with an overall poor prognosis [7]. Bevacizumab has been used to directly target tumor growth by inhibiting the formation of new blood vessels essential for a growing tumor, as shown in Figure 1. By doing so, bevacizumab effectively cuts off the blood supply to cancer, thus eliminating the delivery of oxygen and other essential nutrients to the tumor. As bevacizumab interacts with VEGF, the drug prevents the activation of VEGF receptors on all endothelial cells [8].

When reviewing the effects of bevacizumab within the human body, it is crucial to understand the nature and mechanism of action of this drug. This will allow clinicians to understand how bevacizumab can potentiate severe adverse effects such as sinus venous thrombosis (SVT).

Clinical application of bevacizumab in gliomas

The treatment modalities of gliomas are intricate and differ based on the grade of the tumor. While treatment of high-grade glioma is challenging, low-grade glioma requires little treatment unless it causes mass effects. Due to their highly aggressive and recurrent nature, various treatment approaches have been attempted for rapidly growing gliomas, including surgery, radiotherapy, and chemotherapy. Because of the highly vasculogenic features of high-grade glioma, the use of antiangiogenic drugs has shown to be effective. Among them, bevacizumab is the prime drug that has been experimented with for efficacy and safety and approved for glioblastoma. It is useful alone or in combination with other therapies. Bevacizumab has been endorsed in the United States by the FDA for use in recurrent glioblastoma [9]. Studies have shown that bevacizumab reduces brain edema caused by the abnormal formation of new blood vessels and interruption of the blood-brain barrier. However, the influence on improving overall survival is not significant in newly distinguished glioblastoma when combined with standard therapy, that is, chemoradiation with temozolomide [10-12]. However, phase II clinical trials by Friedman et al. and Taal et al. demonstrated that bevacizumab improves overall survival when used alone or in combination with irinotecan or lomustine in recurrent glioblastoma [13,14]. Normalization of tumor vascularity increasing oxygenation can augment radiotherapy response and distribution of chemotherapeutic drugs [10]. The synergistic effect of radiotherapy and bevacizumab has been shown to reduce radionecrosis and enhance hypofractionated radiation dose without directly affecting the normal brain tissue [15]. In most studies, bevacizumab improved quality of life by displaying a steroid-sparing effect in the case of brain edema.

Recurrence of glioblastoma is higher in CD-133 and Nestin-positive stem cells after chemoradiotherapy [9]. Therefore, anti-angiogenic therapy, such as bevacizumab, is a potent alternative. The development of anti-angiogenic resistance has been a rising issue that needs to be addressed. Multiple hypothesized mechanisms include aggressive tumor growth, non-reliance on VEGF signaling, and rapid proliferation by vasculogenic mimicry, to name a few [16]. Different drug combinations can be the alternative for resistance. In addition, the various side effects of bevacizumab have limited its use in glioma.

Side effects and significant complications of bevacizumab

Bevacizumab is a MAB with VEGF inhibiting properties and is used to cure glioblastoma, advanced colon cancer, breast cancer, and non-small cell pulmonary cancer. The adverse events associated with the usage of bevacizumab include proteinuria (38%), high blood pressure (34%), venous thromboembolism (VT) (17%), arterial thromboembolism (4%), cardiovascular toxicities (4%), bleeding (3-4%), intestinal perforation (2%), and posterior leukoencephalopathy syndrome (<1%) (Figure 1) [17]. Other potential adverse events include delayed wound healing, rashes on the skin, and hypersensitivity reactions related to infusion, for which the patient should be monitored carefully [18].

Nalluri et al. (2008) conducted a meta-analysis of 15 clinical studies employing bevacizumab in a range of primary malignancies to determine the risk of VT in the long run. Patients who received bevacizumab showed a substantially higher chance of VT, with a relative risk of 1.33 when compared to controls [19]. However, considering that VT is a frequent complication in glioblastoma patients (24-30% prevalence), it is unclear if bevacizumab raises the possibility of venous thrombosis in glioblastoma patients [19]. Dural sinus venous thrombosis (DSVT) is a severe complication that can occur due to malignancy. Besides cancer, there are other common risk factors for DSVT such as cytotoxic medication, thrombophilia (genetic or acquired), infection, dehydration, recent surgery, irradiation, and pregnancy [20].

Ozen et al. (2009) published the earliest case of DSVT in a 64-year-old male with metastatic colon cancer who had taken bevacizumab, along with 5-fluorouracil, leucovorin, and irinotecan [21]. Then, in 2011, Vargo et al. described the second case of DSVT in a 25-year-old female with central nervous system malignancy who was managed with bevacizumab, post-surgical radiation treatment, and temozolomide [17]. Thus, clinicians need to keep a track of these occurrences during treatment with bevacizumab, as many of these problems may be prevented by maintaining a careful watch on patient-specific variables [18].

Rate of prevalence, incidence, morbidity, and mortality within the pediatric population

Bevacizumab is considered safe for widespread use. However, clinicians must be aware of the unfavorable complications of bevacizumab, including SVTs. In the pediatric population, there have been more than 40% cases of SVT. Further, the prevalence rate of SVT is 2.6 per 100,000 children per year [22].

The recurrence rate among patients is approximately 70% after six months of their first encounter with SVT. The mortality rate is approximately 10%; however, 17-19% of survivors experience functional defects at discharge. These patients are advised to return to their physician repeatedly to monitor their health status [22-25]. Infants have more than a 50% of mortality rate and poor health outcomes [26].

Sinus venous thrombosis

SVTs are blood clots that form in the prominent veins of the brain, commonly the cavernous sinus, dural sinuses, and deep sinuses of the cortex. These tend to occur in patients with conditions that predispose to clot formation, such as an underlying prothrombotic state or decreased flow within the sinus. Inherited coagulopathies, infections, acute malignancies, and the use of oral contraceptives lead to increased coagulability. The subsequent occlusion of the venous sinuses leads to increased venous pressure due to the decreased cerebral venous outflow. Elevated intracranial pressure is the cause of the signs and symptoms associated with this condition [27].

The most common clinical presentation is headache, although seizures, focal neurological deficits, and altered mental status are also commonly reported. Imaging modalities commonly used to evaluate the condition include MRI and non-contrast CT. However, the sensitivity of these modalities for SVT is low. Magnetic resonance venography and computed tomogram venography, which allow visualization of the thrombus, are preferred. Treatment of the condition includes treating the clot and the predisposing disease which led to clot formation. The former typically involves acute anticoagulation with unfractionated heparin or low-molecular-weight heparin, although thrombolytic therapy may also be required in cases of severe thrombosis. Anticoagulation with warfarin should be continued for three to six months should the thrombosis be provoked and six to twelve months if unprovoked. Current guidelines do not yet recommend direct-acting oral anticoagulants. It has been reported that outcomes of SVTs are typically good if correctly diagnosed and appropriately managed [27].

Bevacizumab in other malignancies

VEGF is expressed in various tumors; however, the proportion of its expression varies within cancer and between different tumors. For example, in glioblastoma multiforme, the tumor is highly expressed adjacent to the necrotic and hypoxic tissue. Similarly, high expression of VEGF is also found in renal cell carcinoma [28]. Studies have also suggested that chemotherapy drugs work better in conjunction with VEGF inhibitors rather than both working alone. Chemotherapy drugs generally damage endothelial cells, and VEGF inhibitors further amplify that effect [29]. The drug has been in trials since 1997 to explore its development toward cancer treatment, and, finally, it was approved for the treatment of metastatic colorectal cancer in 2006 [30].

A phase II clinical trial involving 116 patients concluded that using bevacizumab monotherapy to manage renal cell carcinoma resulted in increased patient survival. The side effects of the treatment remained minimal, including hypertension and asymptomatic proteinuria [31]. A 2007 study reported that the treatment of metastatic renal cancer with bevacizumab and interferon-alpha led to significantly improved progression-free survival [32]. Another study further solidified this improved progression-free survival with combination therapy. However, this study also revealed that side effects such as hypertension, fatigue, and anorexia were significant with combination therapy compared to monotherapy with interferon-alpha [33].

In 2003, a randomized control trial was conducted to test the efficacy and safety profile of bevacizumab as adjuvant chemotherapy for the management of metastatic colorectal cancer. Contrary to the previously mentioned trial for renal cancer, this trial showed that lower-dose bevacizumab had better results than a higher dose [34]. In another phase III trial, a large randomized study was conducted among 923 patients where bevacizumab was used in combination with Irinotecan and 5-fluorouracil. After phase II results, bevacizumab was used at a lower dose (5 mg/kg) for the phase III trial. This combination resulted in a significant overall survival of the patients. Adding bevacizumab did not significantly change the adverse events associated with chemotherapy. However, a few side effects such as epistaxis and grade 3 hypertension were seen at a higher rate. Fortunately, both these adverse events were resolved with standard management options [35].

A phase III trial on ovarian cancer patients included 1,873 women newly diagnosed with stage 3 or stage 4 epithelial ovarian cancer and who had already undergone surgery. In this trial, bevacizumab was used with paclitaxel and carboplatin. The median progression-free survival was 14.1 months in the group that continued bevacizumab throughout the chemotherapy cycles compared to 10.3 months in the control groups. Similarly, mortality was significantly reduced in those who continued bevacizumab throughout the trial. Similar to the colorectal cancer trials, hypertension with grade 2 or higher was commonly seen in the group with bevacizumab therapy. However, this resulted in the discontinuation of bevacizumab only in 15 patients out of the 608 patients in the same group. Thus, it further consolidated that hypertension as a side effect of the therapy is manageable [36]. In another study, the authors reported that, although the previous study showed progression-free survival, the overall survival was not significantly different between all groups. However, on stratification by the stage of cancer, it was found that stage IV cancer patients had a survival advantage with the use of bevacizumab therapy [37]. Antecedently, another study on the ICON7 (International Collaboration on Ovarian Neoplasms) trial emphasized no improvement in overall survival of bevacizumab therapy in conjunction with platinum-based chemotherapy drugs. However, improved survival was witnessed in patients who had much severe disease [38].

Trials to test the effectiveness of bevacizumab in breast cancer management have been conducted. According to the research on metastatic breast cancer, bevacizumab combined with paclitaxel resulted in increased progression-free survival of 11.8 months compared to 5.9 months with paclitaxel alone. However, overall survival was similar for both [39]. Subsequently, another study tested bevacizumab and docetaxel for treating breast cancer with negative human epidermal growth factor receptor 2. The study found higher median progression-free survival with this combination. At the same time, combination with bevacizumab did not impact the adverse outcomes [40]. Finally, the RIBBON-1 (Regimens in Bevacizumab for Breast Oncology) trial showed increased progression-free survival when bevacizumab was combined with capecitabine, taxanes (paclitaxel, docetaxel), or anthracycline (doxorubicin, epirubicin) [41].

In a study on non-squamous cell lung cancer (NSCLC) patients, the addition of bevacizumab to paclitaxel-carboplatin increased survival [42]. A recent study published in 2019 showed that using erlotinib alone to treat epidermal growth factor receptor-positive NSCLC has a median progression-free survival of 13.3 months. On the other hand, combining bevacizumab to the therapy increased it to 16.9 months. However, the combination also resulted in higher grade 3 side effects, with the most common side effect being rash. Grade 4 neutropenia and grade 4 hepatic dysfunction comprised the most severe adverse events [43]. More comprehensive studies are required to understand the survival and adverse events in the patients.

Future recommendation (World Health Organization guidelines)

Bevacizumab is recommended for use in several conditions such as retinopathy of prematurity, colorectal cancer, glioblastoma, renal carcinoma, non-small cell carcinoma, and hepatocellular carcinoma [44]. The dose of bevacizumab used for the above-mentioned conditions varies from 5 mg/kg to 15 mg/kg. It is either used alone or in combination with other chemotherapeutic drugs such as paclitaxel, doxorubicin, carboplatin, and cisplatin. Specific adverse effects documented in the pediatric population were proteinuria, lymphopenia, posterior reverse encephalopathy syndrome, enterocutaneous fistula, epistaxis, dry skin, lacrimation disorder, hypertension, and seizures [45]. It is withheld 28 days before any surgery as it interferes with wound healing. It must be avoided in patients with a prothrombotic state, nephrotic syndrome, or suffering from uncontrolled hypertension. As some patients may have an infusion reaction, necessary precautions must be taken for such patients [44]. Bevacizumab can cause embryo-fetal toxicity, so women who are taking this drug should use effective contraception. Additionally, women taking this drug have a risk of developing ovarian failure. There is a risk of developing congestive heart failure in patients taking this drug. The safety of bevacizumab is not established in the pediatric population (under 18 years of age) as there have been reported cases of non-mandibular osteonecrosis in these populations. The risk of arterial thromboembolism was more significant in patients over 65 years of age using bevacizumab [2]. The risk of hypertension in patients taking bevacizumab is higher in people above 65 years of age than in the younger population.

Furthermore, as bevacizumab is a human MAB, it carries a high risk of infusion reactions. As a precautionary measure, the manufacturer recommends the first infusion of 90 minutes, the second infusion of 60 minutes, and after that 30-minute infusion [20]. As clinicians, it is important to know that bevacizumab should be used with caution in pre-surgical patients, pediatric and geriatric populations, pregnant women, patients with comorbidities, and patients with a history of allergies. Bevacizumab is crucial for treating certain cancers; however, clinicians should be cautious about its use due to its severe side-effect profile.

## Conclusions

Increased incidence of thromboembolic effect post-treatment events with bevacizumab arises most likely due to its anti-VEGF mechanism. Few reports have described an incidence of cerebral SVT in glioma patients receiving bevacizumab. The rate of mortality and morbidity is a matter of concern. Further research and clinical trials are warranted for better guidelines and effective management of cancer patients.
